# Relevance of international partnerships in the implementation of the UN Sustainable Development Goals

**DOI:** 10.1038/s41467-022-28230-x

**Published:** 2022-02-01

**Authors:** Walter Leal Filho, Tony Wall, Jelena Barbir, Gabriela Nagle Alverio, Maria Alzira Pimenta Dinis, Julianna Ramirez

**Affiliations:** 1 Manchester Metropolitan University, School of Science and the Environment, Chester Street, Manchester, M1 5GD UK; 2 Hamburg University of Applied Sciences, Faculty of Life Sciences, Research and Transfer Centre “Sustainable Development and Climate Change Management”, Ulmenliet 20, 21033 Hamburg, Germany; 3 Liverpool John Moores University, Liverpool, L3 5UX UK; 4 Nicholas School of the Environment, Duke University, Durham, NC USA; 5 Sanford School of Public Policy, Duke University, Durham, NC USA; 6 School of Law, Duke University, Durham, NC USA; 7 UFP Energy, Environment and Health Research Unit (FP-ENAS), University Fernando Pessoa (UFP), Praça 9 de Abril 349, 4249-004 Porto, Portugal; 8 CENTRUM Católica Graduate Business School, Pontificia Universidad Católica del Perú, Lima, Perú

**Keywords:** Decision making, Sustainability

## Abstract

To achieve Sustainable Development Goal SDG 17, which focuses on international cooperation, partnerships, will be vital. In this comment, we examine the key obstacles such as vested economic interests that will need to be overcome for the successful implementation of SDG 17.

## Abstract (web summary)

Sustainable Development Goal 17 focuses on partnerships that can enable the achievement of other SDGs. In this comment the authors examine the obstacles to the success of SDG 17 and suggest measures to overcome these.

The implementation of the United Nations Sustainable Development Goals (SDGs) has become ever more important, as the COVID-19 pandemic has exacerbated many existing inequalities. These growing inequalities, especially those observed between industrialised and developing countries hamper equitable sustainable development in both social and environmental terms. For this reason, it is important to recognise the importance of SDG 17—whose focus is on partnerships—as necessary in meeting the other SDGs. SDG 17 advocates for greater cooperation between public, private and third sector organisations, regardless of their origin and size, to implement sustainable development, particularly with developing countries It is of fundamental importance for the continuous development of these associations, and towards the understanding of the role that they play. The complex challenges that society faces on top of climate change, such as the COVID-19 pandemic reminds us of the constant and growing demands that exist, and the urgent need for equitable international partnerships. Combined with good governance, partnerships among international organisations and local and regional associations have the capacity to help to address these growing deficits^1^. Nevertheless, there are many challenges to the successful implementation of SDG 17.

## The role of international partnerships in implementing the SDGs

The role of international partnerships in implementing the SDGs varies according to the context, the organisations, and the specific project needs. Still, the overall goal is to create synergies among the expertise and resource bases of international organisations, Non-Governmental Organizations (NGOs), governments, and private sector institutions, in order to attain the SDGs faster, more efficiently, and more equitably than can be achieved when working in silos^2^. Ultimately, these synergies are meant to create win–win situations in which all partners achieve both, individual and common goals^3^. Bull and McNeill^4^ identify five major types of international partnerships: local implementation, resource mobilisation, advocacy, policy development and lobbying, and market-based operations. Beyond these major roles, international partnerships also have the potential to push the SDGs agenda forward through advancing positive rules, creating cross-sectoral connections at the policy level, shifting individual and organisational behaviour, and empowering marginalised and vulnerable people^5^.

In practice, partnerships can take a variety of approaches based on the specific skills each member brings, and the needs they seek to address. They can focus on just one SDG, or take a systematic approach that addresses several of them at the same time. The Positive Impact Events international partnership, for instance, tackles themes across 12 SDGs by establishing and sharing inclusive knowledge-sharing best practices so that the reach of SDG-related events is at least 1.5 billion people per year, whereas Objectif 2030 is a global project that solely addresses SDG 17 (Table 1). Although it is impossible to assert that these initiatives would not have occurred without international partnerships in place, it is likely that they would have taken much longer to be organised and could lead to smaller-scale results. It is concluded that partnerships clearly contribute to the commitment of society to global sustainability, fostering economic growth, empowering people, and simultaneously focusing on sustainable development, as illustrated in the examples given in Table 1.

**Table 1:** Ten selected examples of partnerships implementing the SDGs, data from United Nations (2021)^12^.

Partnership	Aims and countries involved	SDGs
Global Voices SDG Youth Fellowship (The Dais) www.thedais.in	Supports the university students to work on the effective implementation of the program and showcase the impact that young people can have in the development, social and professional space, finding their own expertise, space, and place in the implementation of the UN 2030 Agenda. (3 countries)	
Engaging the global event sector in the UN SDGs (Positive Impact Events) www.positiveimpactevents.com	Increases the capacity to deliver actions to achieve the SDGs so that at least 1.5 billion people a year attend events, acquire knowledge and skills to promote sustainable development. (58 countries)	
Creating Sustainable Means to Empower the Vulnerable in Rural Senegal (Alfity ANU Humanitaire International) http://alfityanu.org/	Empowers disabled women, widows, orphans, and other vulnerable groups within the population in rural Senegal. Aims to improve food and nutrition situation, access to education and medication in a long term, and strengthening position within the community. (2 countries)	
Legal and Economic Empowerment Global Network (LEEG-net) http://www.leeg-net.org/	Fosters legal innovation and empowerment of the poor and disadvantaged groups in meeting the “greatest global challenge of eradicating poverty”, considered the unifying thread throughout the 17 Goals, 169 targets, and 232 indicators. (5 countries)	
YouthCan! Global partnership for youth employability (Decent Jobs for Youth) https://www.decentjobsforyouth.org/commitment/114	Supports disadvantaged young people to successfully manage the transition from school to independent adulthood. (31 countries)	
Objectif 2030 http://www.objectif2030.org/	Seeks to address the need for comprehensive information on sustainable development and to support innovative actions and solutions that combine social inclusion, economic growth, and nature protection. (Global scope)	
Accessible for People with different ability: Tourism and Education (International Development Institute) https://idiworldwide.net/	Commits to design, develop, and implement capacity-building programs for accessibilities in Nepal. Focus on reducing discrimination against people with different abilities for tourism and also to reduce the gender digital divide in education. (1 country)	
Assisting with disaster relief mapping for medical, energy, and climate erosion damages https://github.com/wgdesign/un-projects	Understands and develops a rapid response to climate change relief through mapping and advanced data analysis and technology, either in disaster scenarios or climate change. (3 countries)	
Partnership for Action on Green Economy (PAGE) https://2019.page-annual-report.org/	Seeks to put sustainability at the heart of economic policies and practices to advance the UN 2030 Agenda and supports nations and regions in reframing economic policies and practices around sustainability to foster economic growth, create income and jobs, reduce poverty and inequality, and strengthen the ecological foundations of economies. (20 countries)	
World Social Capital Monitor (Trust Your Place) https://www.trustyourplace.com/	Promotes social goods, i.e., trust, solidarity, helpfulness, friendliness, and hospitality, an interdisciplinary non-material core asset to achieve the SDGs. The Open Access Software allows to identify, assess, enhance and encourage local social goods. (117 countries)	

Just as the need for international partnerships is growing, the urgency of the problems they will seek to address is also increasing. Climate change, COVID-19 pandemic recovery and preparedness, political polarisation, conflicts, and economic inequality, to name a few, are pressing issues whose causes and solutions reinforce one another. Whereas they are global in nature and often have slow onsets, addressing them often requires swift actions. Long-term sustainable international partnerships should be exploited as drivers of change for addressing these challenges and transforming society^6^.

## Obstacles in the implementation of SDG 17

The challenges in implementing SDG 17 are significant, exacerbated by the SDGs being all-encompassing without clear responsibility and accountability^7^. One of the most fundamental challenges is *economics*. The growth and stabilisation of economies often take precedence over the pursuit of SDGs at the national policy level hence negatively affecting the engagement of private companies in SDGs collaborations^8^. For example, some nations whose economies are dependent on tourism, have adopted trade policy and partnerships which promote tourism for its economic benefits but do not address the related impacts of increased levels of travel and tourism on local efforts to mitigate local climate change^9^.

These imbalances of power can be especially problematic for low-income countries, whose partnership agendas are mostly influenced by developed nations^8^. For example, large internationally funded infrastructure projects in Africa, such as building a new expressway in Uganda, are typically driven by economic wealth to the detriment of local communities who have been left socially and economically stranded and whose land has been swamped with pollution. A measure that could address this is local stakeholder involvement in these internationally funded projects at an early planning stage to ensure that impacts are minimised and that the benefits are maximised. Other measures include giving employment to local people so they can see the advantages of the project.

Reconciling different, and potentially oppositional perspectives is also a significant challenge^10^. This is partly because of the different levels of involvement, understanding, and commitment to the various SDGs by different stakeholders involved^7^. Such complexities in partnerships can create tensions when an existing partner’s attitudes or organisational or political preferences are focused on short-term outcomes, such as the completion of a project rather than extensive consultation and participatory decision making, despite the need for a longer timeframe for accomplishing goals. Similarly, international partnerships which focus on a single SDG, or a small group of them, may well unintentionally negatively affect the achievement of a different SDG which is important to one of the international partners^9^. For example, the Vietnamese government has spent decades developing access for ethnic minority communities into work (a short-term employment outcome) with support from international agencies. However, these partnerships have not sufficiently considered wider climate issues, so the promotion of clean and green industries remains stagnant and key skills are predominantly sourced overseas. A measure that can address this is the preparation of integrated sustainable development plans, which analyses the actual or possible impacts on all SDGs for a range of sectors and communities^10^.

Engagement at the local level is also a further, practical challenge. When designing and delivering partnerships for the SDGs, it is important to use approaches that enable local people to participate in the process. Evidence tells us that there are still challenges in engaging important marginalised groups and integrating localised and indigenous knowledge of sustainability into partnership works^7^. A measure to overcome these challenges includes designing equitable partnerships to enable a wide diversity of people to participate, and these approaches are then shared to build capacity for all involved^10^. Collaborative approaches include ways of working together which enable genders, cultures, castes, or socio-economic groups to work together, even if typical cultural settings would not normally support these ways of working.

Institutional and legal barriers may also pose an obstacle in respect of international partnerships, where different bureaucratic or commercial protectionism can significantly hinder collaborative efforts. This can be particularly observed within partnerships attempting to promote clean and affordable energy. For example, where there are major challenges in accessing and sharing data and sometimes equipment-related to sustainable energy use. Data are often heavily guarded by oligopolies, which place a great financial value on it, but also often have responsibilities in managing the safety, security, and resilience of power networks across large geographic networks and regions^11^. Political leadership supporting sustainable development at the highest levels is a critical measure for addressing this challenge^10^. This can include politicians setting policies that state ambitious targets or tax arrangements that incentivise their achievements, such as tax benefits or lower business charges for lower business or transport emissions.

## Outlook

There are encouraging signs of progress in respect to the implementation of SDG17, but the current obstacles addressed in this commentary are hindering progress. One way forward is by placing SDG17 more prominently as a cross-cutting SDG. Clear processes and structures should be put in place to encourage partnerships at both local and global levels. And that developing countries are better placed- to take better advantage of the many opportunities they offer, exploring available resources. The key to achieve this is for companies, organisations, and governments to create alliances at all levels, especially in relation to human and logistical resources, economic development, and access to new technologies and social innovation. Some of these actions include responsible international trade with shared value policies aimed at the SDGs beyond legal obligations. In doing so, this means that more strategic alliances are needed in order to achieve synergies and maximise the use of those resources, as well as intending to prevent the duplication of efforts.

As this commentary has shown, the worldwide implementation of the SDGs is presented as a dichotomy between the many threats to their implementation and the opportunities that exist in building momentum for them, particularly in the COVID-19 post-pandemic world we find ourselves in. The SDGs are a roadmap to the year 2030 that not only invites, but also requires organisations to work together and undertake specific actions to address the many challenges the world is facing today. The SDGs can only be fully achieved by means of global, regional, and local partnerships, where both the private and public sectors work together with NGOs, charities, stakeholder groups, and funding agencies.
